# Physical Activity, Metabolic Risk and the Primary Allostatic Load Mediators: An Explorative Study

**DOI:** 10.3390/sports14030107

**Published:** 2026-03-09

**Authors:** Francis Osei, Pia-Maria Wippert, Andrea Block

**Affiliations:** 1 Medical Sociology and Psychobiology, Department for Physical Activity and Health, University of Potsdam, 14496 Potsdam, Germany; wippert@uni-potsdam.de (P.-M.W.); andrea.block@uni-potsdam.de (A.B.); 2 Faculty of Health Sciences, Joint Faculty of the University of Potsdam, The Brandenburg Medical School Theodor Fontane, and the Brandenburg University of Technology Cottbus-Senftenberg, Am Mühlenberg 9, 14476 Potsdam, Germany

**Keywords:** allostatic load, exercise training, metabolic syndrome, primary mediators, stress

## Abstract

Background: Chronic stress is associated with dysregulation of the body’s allostatic systems, contributing to increased allostatic load (AL) and adverse metabolic outcomes. Regular physical activity (PA) is considered a key protective factor that may attenuate AL by enhancing adaptive stress responses and supporting metabolic health. This study examined the differences between PA, primary mediators of AL, and metabolic risk markers in apparently healthy adults in Germany. Methods: Forty-six adults (18–45 years) were categorized into a moderate intensity (regular PA: ≥150 min a week vs. non-regular PA: ≤150 min a week) group according to current PA recommendations. Primary AL mediators were quantified by cortisol (μg/12 h), epinephrine (μg/12 h), norepinephrine (μg/12 h), and dehydroepiandrosterone sulfate (DHEA-S: μg/mL). Group differences in primary AL mediators and metabolic risk markers were examined using the Mann–Whitney U test. Results: A significant group difference was observed for cortisol levels, with higher values in the regular PA group (*p* = 0.01), with a moderate negative effect size of *r* = −0.38. No statistically significant differences (*p* > 0.05) were found between groups for epinephrine, norepinephrine, DHEA-S, or metabolic risk markers, including triglycerides, blood pressure, body mass index (BMI), and high-density lipoprotein cholesterol (HDL-C). Conclusions: The findings suggest that regular PA may be associated with altered stress-regulatory activity, as reflected by differences in cortisol. While no statistically significant group differences were observed for metabolic risk markers, descriptive patterns indicate more favorable lipid profiles and potential variation in primary AL mediators at higher PA levels. Given the exploratory nature of the analyses and the small and unequal group sizes, these findings should be interpreted with caution and warrant confirmation in future studies with larger and more balanced samples.

## 1. Introduction

Stress affects millions of people worldwide. The increasing prevalence of stress may be attributed to multiple factors, including environmental stressors, socioeconomic pressures, and unhealthy lifestyle changes [1,2]. Acute stress activates the sympathetic nervous system (SNS), leading to the release of epinephrine and norepinephrine and initiating the fight-or-flight response [3,4]. In contrast, chronic or prolonged stress predominantly activates the hypothalamic–pituitary–adrenal (HPA) axis [5,6]. This process involves hypothalamic release of corticotropin-releasing hormone (CRH), which stimulates adrenocorticotropic hormone (ACTH) secretion from the pituitary and subsequently promotes cortisol release from the adrenal glands [7,8,9].

While acute stress responses are essential for physiological functioning and adaptation, chronic stress disrupts the body’s regulatory systems and contributes to cumulative physiological strain [10]. This adaptive process, termed “allostasis”, describes how the body maintains stability through change [11,12]. However, prolonged or repeated exposure to stress results in cumulative “wear and tear” on physiological systems, a concept known as allostatic load (AL) [11,13,14]. Additionally, AL can be assessed using biomarkers across multiple physiological systems, with primary mediators reflecting immediate neuroendocrine responses to stress. These primary mediators include cortisol, epinephrine, norepinephrine, and dehydroepiandrosterone sulfate (DHEA-S) [15,16,17,18,19,20]. Chronic stress and elevated AL have been associated with the development of stress-related disorders and metabolic diseases, including metabolic syndrome (MetS) [1,21,22].

MetS is characterized by a cluster of cardiometabolic abnormalities, including elevated blood pressure, abdominal obesity, reduced high-density lipoprotein (HDL) cholesterol, increased triglycerides, and hyperglycemia [22,23]. Cortisol plays a central role in regulating glucose metabolism, immune function, and blood pressure. However, chronic elevations are associated with visceral adiposity, insulin resistance, and hypertension [24,25]. Similarly, sustained SNS activation, reflected by elevated epinephrine and norepinephrine levels, increases cardiometabolic risk through heightened heart rate, blood pressure, and vascular resistance [26,27]. DHEA-S acts as a counter-regulatory hormone that buffers the effects of cortisol, yet its levels often decline under chronic stress, further contributing to metabolic dysregulation [28,29].

Exercise is a subset of planned and repetitive physical activity (PA) performed to improve physical fitness and maintain health [30]. PA can be categorized into two types: exercise, which refers to engaging in high-intensity PA akin to that of an athlete (e.g., a minimum of 10 h per week of training), and leisure sports, which involves moderate PA during recreational or leisure time [31]. Acute exercise acts as a stressor that temporarily activates the hypothalamic–pituitary–adrenal (HPA) axis and sympathetic nervous system (SNS), resulting in transient increases in cortisol and catecholamines [24,32]. In contrast, regular endurance exercise and high-intensity interval training (HIIT) promote long-term adaptation, reducing resting levels of stress hormones and enhancing the efficiency of stress-responsive systems, possibly through enhanced catechol-O-methyltransferase (COMT) activity [24,33]. Collectively, regular PA supports improved stress recovery and physiological resilience by reducing HPA axis hyperactivity and lowering excessive SNS activation [34,35]. These adaptive mechanisms include better autonomic balance, increased endorphin release, and enhanced neuroplasticity [36], helping modulate AL and maintain metabolic regulation [37,38].

To better understand the pathophysiological mechanisms linking PA, AL, and metabolic risk, research in populations without diagnosed stress-related or metabolic diseases is warranted. The majority of AL research to date has focused on diseased populations, including individuals with depression or elevated AL, who often present with subclinical metabolic abnormalities such as impaired glucose tolerance or borderline hypertension that may progress to MetS [22,39,40,41,42]. In Germany, prior studies have demonstrated associations between MetS and stress-related conditions such as depression across age groups [40,41]. Exercise training has been shown to reduce MetS severity, depressive symptoms, and key metabolic risk markers, including body mass index (BMI), systolic blood pressure, and waist circumference [43].

Despite evidence linking PA, AL, and metabolic risk, limited research in German populations has examined these associations in apparently healthy adults. This study addresses this gap by examining differences between PA, primary AL mediators, and metabolic risk markers in an apparently healthy German adult population.

## 2. Materials and Methods

### 2.1. Study Design

The analysis is based on data from parallel study 3 (i.e., PSA 3 study) within the National Research Network, “Medicine in Spine Exercise (MiSpEx) Network” [44,45]. The PSA 3 was a study on psychometric and biological measures in adults. The PSA 3 was a longitudinal observational study with measurement time points (M1–M4) taken every four months between August 2013 and June 2015 [45,46]. The PSA 3 study data were collected using standardized questionnaires (M1–M4) and biomarkers from hair, blood, and urine (M1, M4), with laboratory assessments conducted by trained nurses. For collection procedures for urine and blood samples, check [45,46]. For the current study, a subsample (*n* = 46) of the baseline data (M1) was used. Additionally, the parent longitudinal design improved data quality and internal validity by ensuring that all measurements were gathered using consistent, protocol-driven techniques [45]. The baseline analysis offers a methodologically sound snapshot of early physiological dysregulation. This is in line with the study’s aim of characterizing preclinical associations in people without any identified metabolic disorder, even though longitudinal analyses were outside the purview of this current investigation.

### 2.2. Study Population

Participants were recruited from the Ernst von Bergmann clinic and the University of Potsdam outpatient clinic, with a final sample of *n* = 140 individuals aged 18 to 45 years included in the study. As an incentive, participants received their clinical laboratory findings and a personalized stress profile upon study completion [45]. The participants included in this current study were able to complete a German questionnaire and had at least one episode of non-specific low back pain (LBP) lasting four or more days within the previous 12 months. As no diagnosed cardiovascular or metabolic diseases were present [45], participants were classified as “apparently healthy” within the context of the present study. Exclusion criteria were pregnancy, acute infections, hormone therapy, or the use of specific medications (such as glucocorticoids and antibiotics); certain diseases (such as cardiovascular, metabolic, thyroid, vascular, malignant, lung, or autoimmune diseases); hemophilia; and psychological disorders (such as those listed in ICD-10: F70–79) and hair shorter than 2 cm [45]. For the present analysis, a subsample of *n* = 46 who had completed the AL biomarker assessment was used [45].

### 2.3. Ethical Approval

The study was carried out in compliance with the values outlined in the Declaration of Helsinki of 1975, as revised in 2013. A study nurse provided written and spoken information about the study to each participant before they all signed the written informed consent form. The main institutional ethics review board of the University of Potsdam, Germany, granted the final ethical approval on 6 May 2013 (No. 44/2012).

### 2.4. Physical Activity Assessments

A self-structured PA questionnaire was designed to capture weekly frequency and durations of PA domains, consistent with World Health Organization (WHO) PA guidelines [31]. High-intensity PA (labeled as exercise) was defined as above 10 h a week, and leisure sport was defined as below 10 h a week within the MiSpEx Network [44]. The instruments were tested for reliability and validity in several pilot studies conducted in the context of developing multiple randomized controlled trials before being implemented. The MiSpEx network randomized controlled trials were registered as DRKS00004977 [47] and DRKS00010129 [48] in the German Clinical Trials Register. Leisure sports were calculated as the product of time (min) and days spent performing activities such as walking, stair climbing, and cycling. Similarly, exercise was calculated as the product of time (min) and days spent performing exercise training (e.g., football, judo, swimming, basketball, etc.). Furthermore, an additional scale was calculated for the total PA (PA, min/wk), which was the product of days and time (min) spent performing both exercise and leisure sports. Based on total PA, study participants were categorized into two groups: regular PA (*n* = 37, ≥150 min/week) and non-regular PA (*n* = 8, ≤150 min/week) groups in accordance with WHO recommendations for health promotion. This categorical approach was performed to reduce sensitivity to recall error and bias and thus align exposure classification with established public health thresholds [31].

### 2.5. Primary Mediators of Allostatic Load Assessment

12 h urinary cortisol (μg/12 h) was assessed via enzyme-linked immunosorbent assay (ELISA, RE52241, IBL International GmbH, Hamburg, Germany). Serum DHEAS (μg/mL) was assessed via ELISA (RE52181, TECAN Hydro Flex, IBL International GmbH, Hamburg, Germany) [45]. A 12 h overnight urinary epinephrine (μg/12 h) and norepinephrine (μg/12 h) levels were assessed via ELISA (epinephrine RE59251, norepinephrine RE5926, both by IBL International GmbH, Hamburg, Germany) [45]. Cortisol, epinephrine, norepinephrine, and DHEA-S were computed to calculate the primary allostatic load index (ALI). The primary (ALI) was constructed using a sample-based quartile, consistent with established methodology [45]. The AL biomarker values in the fourth quartile (>75%) of the parent cohort distribution were given a value of “1” (indicating relatively higher physiological burden), while values below this threshold were given a value of “0” (unburdened) [45].

### 2.6. Blood Pressure Measurement

Outpatient nurses measured systolic and diastolic blood pressure at three different times, with a 30 s rest interval in between each measurement. The readings of the second and third were averaged to provide the final blood pressure readings. The measurement was taken with a BOSO BS 90 Blood pressure device (BOSCH + SOHN GmbH u. Co., KG, Jungingen, Germany) [45,46].

### 2.7. Lipid and Glucose Metabolic Biomarkers

Using enzymatic colorimetric assays (ABBOTT Architect ci8200; Abbott Laboratories, Abbott Park, IL, USA), triglycerides, HDL-C, and LDL-C were measured. In addition, measurements of height (Seca 222 telescopic measuring rod; Seca AG, Reinach, Switzerland), weight (Kern MPS scale; Kern & Sohn GmbH, Balingen, Germany), and waist/hip circumference (customary measuring tape) were taken [46]. The waist circumference was measured above the umbilicus at the narrowest point between the ribs and the iliac crest, and the hip circumference was measured at the widest point across the buttocks. The ratio of weight (kg) to height (m^2^) was calculated for body mass index (BMI) [45,46]. Glycosylated hemoglobin (HbA1c) was measured using high-performance liquid chromatography (HPLC) Bio-Rad Variant II (Bio-Rad Laboratories, Hercules, CA, USA); fasting insulin was measured using an electrochemiluminescence enzyme immunoassay (ECLIA) using a Roche Cobas 8000 Modul E620 (Roche Diagnostics Ltd., Basel, Switzerland); and fasting glucose was measured by a hexokinase enzymatic reaction using a Roche Cobas 400 Plus (Roche Diagnostics Ltd., Basel, Switzerland) [45]. The following formula was used to compute insulin resistance [using the Homeostasis Model Assessment Index (HOMA)]: glucose [mg/dL] × insulin [mU/mL]/405 [45].

### 2.8. Statistical Analysis

Data was analyzed using the Statistical Package for the Social Sciences (SPSS 29.0, Chicago, IL, USA) computer software. All primary mediators and metabolic risk markers were analyzed as continuous variables in group comparisons.

Normality was assessed using the Shapiro–Wilk test. As the distribution was skewed, a non-parametric statistical method was employed, as it is less sensitive to distributional assumptions and unequal group sizes. Descriptive data are presented in Table 1. Group comparisons between PA categories regarding primary mediators of AL and metabolic risk markers were conducted using the Mann–Whitney U test. The Mann–Whitney U test preserves rank-order information and does not require dichotomization of continuous data. This test was selected for its robustness for small and unequal sample sizes. Also, given the small and unequal sample size constraints, adjustment for multiple comparisons was not conducted. The analysis was rather considered exploratory and focused on effect direction and magnitude rather than inferential generalization. Adjustments for covariates such as sex, age, and BMI were not performed due to the small sample and unequal group sizes. This would have substantially increased the risk of model overfitting and unstable estimates. Additionally, all missing datasets were excluded from the final analysis. The statistical significance level was set at *p* < 0.05.

## 3. Results

Finally, a total of *n* = 46 participants were included in the study (65.2% female, 32.6% male; median age = 30 years). Among them, 37 participants (80.4%) engaged in regular PA, and 97.4% additionally participated in leisure sports activities. One participant reported being physically active only through leisure sports. Regarding the biomarkers, all participants were within recommended normal or physiological ranges, such as for cortisol (μg/12 h), epinephrine (μg/12 h), norepinephrine (μg/12 h), and DHEA-S (μg/mL). Applying metabolic risk markers, *n* = 9 (19.6%) of the sample were identified with elevated waist circumferences, *n* = 4 (8.7%) with elevated blood pressure, *n* = 2 (4.3%) with elevated blood glucose, *n* = 2 (4.3%) with elevated triglycerides, and an additional *n* = 2 (4.3%) with reduced HDL-C.

**Figure 1:**
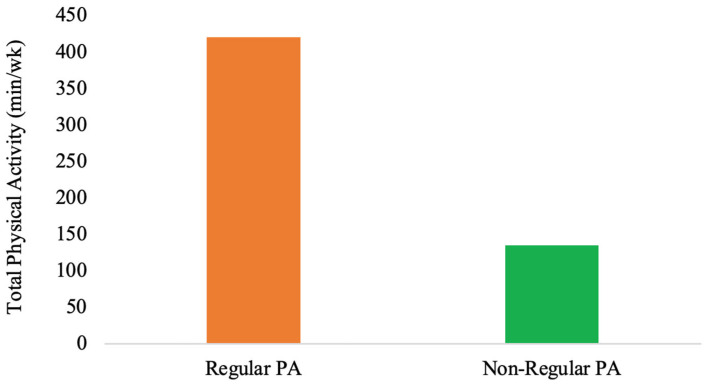
Differences in Total physical activity (min/wk) between regular PA and non-regular PA participants.

Group comparisons indicated differences between individuals who engage in regular PA and those who incorporate PA only occasionally into their daily routines (non-regular PA). Regularly active individuals exhibit approximately a fourfold higher level (see Figure 1) of activity (regular PA: *Mdn* = 420 min vs. non-regular PA: *Mdn* = 135 min; *U* = 35.500, *Z* = −2.648, *p* = 0.008). The higher physiological strain in this group is reflected in the primary AL mediator cortisol (regular PA: *Mdn* = 130.60 μg/12 h vs. non-regular PA: *Mdn* = 71.50 μg/12 h; *U* = 61.00, *Z* = −2.583, *p* = 0.01) with a moderate negative effect size of *r* = −0.38 (effect size calculation not included in Table 2). While cortisol differed between the groups, no corresponding significant differences (*p* > 0.05) were observed in metabolic risk markers such as triglycerides, blood pressure, waist circumference, fasting blood glucose, BMI, WC, and HDL-C between the two groups (see Table 2). No significant differences were observed between epinephrine, norepinephrine, and DHEA-S.

## 4. Discussion

The present study investigated the different PAs on the primary mediators of AL and metabolic risk markers in apparently healthy adults. Individuals who engaged in regular PA demonstrated an approximately fourfold higher activity level compared to the non-regular PA group. In the regular PA group, the total PA (min/week) was (*Mdn* = 420 min/week). The current descriptive findings align with these data and underscore the preventive relevance of maintaining consistent PA across adulthood.

Additionally, the higher physiological strain in the regular PA group is reflected in significantly elevated cortisol levels, as well as slight increases in epinephrine and norepinephrine levels. Although regular PA is often associated with improved stress regulation [24,33,37,38], elevated cortisol in this context may be a reflection of adaptive neuroendocrine engagement rather than pathological dysregulation [7]. Notably, the repeated stress from exercise training frequently activates the HPA axis, leading to ACTH hypersecretion due to adrenal enlargement, which triggers cortisol release. On one hand, this can result in higher basal and exercise-induced cortisol levels as the body becomes more efficient in responding to stress [24,49]. On the other hand, excessive and high-intensity exercise training without adequate recovery may lead to overtraining syndrome, which results from prolonged HPA axis activation and disrupted circadian rhythms [50,51]. In this current study, a more detailed analysis of the descriptive data reveals that the presented cortisol levels may be influenced by the volume and intensity of exercise. This finding aligns with other studies, which have reported higher cortisol levels in individuals who engage in exercise training compared to those who do not participate in exercise training [24,50]. This phenomenon can be attributed to the exercise-induced glucocorticoid paradox, where elevated cortisol may reflect an adaptive neuroendocrine activation instead of pathology [52]. Interestingly, within the AL framework, higher cortisol does not necessarily indicate harm but may signal sustained HPA axis activation in response to repeated physiological demands [7]. In this sample, regular PA, particularly at higher volumes (~420 min/week), may impose cumulative stress when recovery, sleep, or psychosocial context are not accounted for. This warrants cautious interpretation of the current findings.

In comparing the two groups (i.e., regular PA vs. non-regular PA), no significant group differences were observed for epinephrine, norepinephrine, primary ALI, and DHEA-S. Interestingly, similar results have been found in other studies [53,54]. It should be noted that during both acute stress and exercise, epinephrine is released into the bloodstream from the adrenal medulla as a result of SNS activation [32]. This triggers the body for energy mobilization by stimulating lipolysis, hepatic glucose output, and suppression of insulin secretion through the β-adrenergic signaling pathways [34,55]. In physically active individuals, acute or moderate increases in epinephrine have been shown to be associated with enhanced metabolic flexibility and reduced central adiposity [56,57]. Additionally, regular PA improves norepinephrine activity and function resulting from an adaptive response to the HPA axis and catecholamines by the activity of COMT [24,33,58].

Epinephrine enhances fat oxidation and thermogenesis, resulting in lower visceral fat accumulation, which is one of the key components of MetS [59]. Long-term PA participation not only temporarily increases epinephrine levels during exercise but also improves β-adrenergic sensitivity and mitochondrial efficiency in skeletal muscle. This contributes to better glucose absorption and insulin sensitivity [60]. As described in the stress physiology framework, catecholamines serve as primary mediators of allostatic responses [26,27]. Importantly, the observed catecholamine patterns in this current study are interpreted within the AL framework. Both urinary epinephrine and norepinephrine serve as indicators of neuroendocrine activity associated with physiological stress regulation [44,45]. The findings reflect neuroendocrine activity at the time of assessment that may be associated with regular PA participation. The findings are consistent with prior research suggesting that PA may be associated with altered catecholamine signaling linked to metabolic regulation, including reduced insulin antagonism and enhanced lipolytic processes [56,57]. However, these associations should be interpreted cautiously, as stress responses to PA vary depending on exercise intensity, duration, and modality [24,33,58], and causal relationships cannot be inferred due to the cross-sectional design of the current study.

Anthropometric measures such as BMI and waist circumference did not differ significantly between groups. However, the regular PA group showed numerically lower triglyceride levels and higher HDL-C concentrations compared with the non-regular PA group. This pattern is consistent with existing evidence linking PA to improved lipid metabolism, even in the absence of statistically significant differences in lipid and glycemic markers [61]. In line with studies demonstrating vascular and autonomic benefits of regular exercise, both groups exhibited similarly low systolic and diastolic blood pressure values [62]. Together, these findings support the notion that regular PA contributes to the maintenance of metabolic homeostasis through neuroendocrine and cardiovascular pathways.

Within the AL framework, this pattern may reflect early compensatory physiological responses rather than established metabolic dysregulation. From a theoretical perspective, prolonged stress exposure can lead to cumulative physiological dysregulation across immunological, metabolic, and neuroendocrine systems [12,13,14,63]. PA represents a key behavioral regulator of these processes. Exercise enhances systemic flexibility and reduces basal allostatic load by introducing acute, manageable stressors that recalibrate sympathetic nervous system activity and the HPA axis [37,38]. These adaptations may mitigate the development of MetS by improving insulin sensitivity, lipid turnover, and endothelial function [29,36,64,65].

Nevertheless, elevated blood glucose, waist circumference, triglycerides, and reduced HDL-C were observed in a subset of participants, indicating that subclinical cardiometabolic risk markers can be present even in apparently healthy individuals and may contribute to later development of MetS [42]. Accordingly, preventive strategies—including regular PA and routine medical check-ups—remain important even in healthy populations, particularly to identify and manage latent risk profiles such as genetically predisposed lipid metabolism abnormalities.

The current study has limitations that should be considered by future researchers. This was a secondary data analysis. Thus, the reliance on self-reported PA, the small sample size, gender imbalance favoring females, and the cross-sectional sample limit causal inference, analysis methods, and generalization. Additionally, the absence of correction for multiple comparisons may increase the risk of type I error [66]. Accordingly, the results should be interpreted as exploratory and hypothesis-generating. Although relevant covariates such as age, sex, diet, sleep, BMI, and timing of cortisol sampling were available in the dataset, adjustment for these variables was not performed due to the small and unequal subsample size, which would have substantially increased the risk of model overfitting and unstable estimates. Therefore, causal interpretation of the observed associations is limited, and future studies with larger samples are warranted to confirm these findings [67,68,69]. Also, information concerning the number of years the participants have been participating in PA was unknown; hence, care should be taken when interpreting the results. Future research should replicate these findings with larger, longitudinal cohorts and multimethod PA assessments to enhance validity. Additionally, there are different scoring procedures and biomarkers used in calculating primary ALI [17,19]. This could lead to different outcomes. In the current study, ALI was calculated from [70]. However, assessing AL across the various neuroendocrine systems is complex and warrants further research.

## 5. Conclusions

In conclusion, findings from this exploratory investigation indicate that regular PA may be associated with altered stress-regulatory activity, as reflected by higher cortisol levels alongside stable secondary metabolic risk markers. Differences in triglycerides and HDL-C were observed numerically, but group differences in metabolic risk markers were not statistically significant. However, given the cross-sectional design, small sample size, and methodological limitations, these findings should be interpreted as exploratory and hypothesis-generating. Future longitudinal studies with larger samples and with adequate power are needed to clarify how sustained PA influences neuroendocrine function, allostatic processes, and long-term metabolic health.

## Figures and Tables

**Table 1:** Descriptive statistics of study participants (*n* = 46, 100%).

Variables	*N*	%	Median	IQR	Range
**Age (years)**	45	97.8	30.00	15.00	25.00
**Sex**					
Male	15	32.6	-	-	-
Female	30	65.2	-	-	-
**Primary mediators of AL**					
Cortisol (μg/12 h)	46	100.0	113.55	73.70	238.60
Epinephrine (μg/12 h)	46	100.0	5.95	5.30	16.80
Norepinephrine (μg/12 h)	46	100.0	24.40	20.60	95.50
DHEA-S (μg/mL)	46	100.0	0.53	0.89	2.48
Primary ALI	46	100.0	1.00	2.00	4.00
**Anthropometry**					
BMI (kg/m^2^)	44	95.7	22.29	3.13	13.07
Waist circumference (cm)	45	97.8	75.20	13.00	42.30
**Blood pressure**					
Systolic blood pressure (mmHg)	45	97.8	100.00	13.75	50.00
Diastolic blood pressure (mmHg)	45	97.8	60.00	10.00	35.00
**Lipid profile**					
Triglycerides (mg/dL)	46	100.0	89.90	48.90	129.80
HDL-C (mg/dL)	46	100.0	62.25	17.60	59.50
**Glycemia**					
Fasting blood glucose (mg/dL)	46	100.0	86.30	7.40	29.40
**Lifestyle habits**					
Leisure Sports (min/week)	38	82.6	150.00	225.00	460.00
Exercise (min/week)	37	80.4	240.00	345.00	1275.00
Total Physical Activity (PA) (min/week)	43	93.5	330.00	453.00	1680.00

Key: DHEA-S: dehydroepiandrosterone sulfate; Primary ALI: primary allostatic load index; BMI: body mass index; HDL-C: high density lipoprotein cholesterol; *N*: number; %: percentage; IQR: interquartile range; PA: leisure sports; Exercise: high-intensity training more than 10 h a week; Total Physical Activity: sum of PA and exercise.

**Table 2:** Differences between Anthropometric and biomarkers between regular PA and non-regular PA groups.

Variables	Regular PA (*n* = 37)				Non-Regular PA (*n* = 8)				U	Z	*p*
	N (%)	Mdn	Mean Rank	Sum of Mean Ranks	N (%)	Mdn	Mean Rank	Sum of Mean Ranks			
**Age**	37 (80.43)	28.00	22.07	816.50	8 (17.40)	37.50	27.31	218.50	110.500	−1.026	0.30
**Sex**											
Male	14 (30.43)	-	-	-	1 (2.17)	-	-	-	-	-	-
Female	23 (50.00)	-	-	-	7 (15.22)	-	-	-	-	-	-
**Anthropometry**											
BMI (kg/m^2^)	35 (76.08)	22.34	22.63	792.00	8 (17.40)	21.92	19.25	154.00	118.000	−1.694	0.49
Waist circumference (cm)	36 (78.26)	75.60	22.42	807.00	8 (17.40)	79.10	22.88	183.00	141.000	−0.091	0.92
**Primary Mediators of AL**											
Cortisol (μg/12 h)	37 (80.43)	130.60	25.35	938.00	8 (17.40)	71.50	12.13	97.00	61.000	−2.583	0.01
Epinephrine (μg/12 h)	37 (80.43)	7.00	24.27	898.00	8 (17.40)	4.40	17.13	137.00	101.000	−1.396	0.16
Norepinephrine (μg/12 h)	37 (80.43)	25.30	23.89	884.00	8 (17.40)	19.00	18.88	151.00	115.000	−0.980	0.32
DHEA-S (μg/mL)	37 (80.43)	0.50	22.38	828.00	8 (17.40)	0.69	25.88	207.00	125.000	−0.683	0.49
Primary ALI	37 (80.43)	1.00	23.41	866.00	8 (17.40)	0.50	21.13	169.00	133.000	−0.473	0.63
**Blood Pressure**											
Systolic blood pressure (mmHg)	36 (78.26)	100.00	21.68	780.50	8 (17.40)	102.50	26.19	209.50	114.500	−0.909	0.36
Diastolic blood pressure (mmHg)	36 (78.26)	60.00	22.36	805.00	8 (17.40)	60.00	23.13	185.00	139.000	−0.167	0.86
**Lipid Profiles**											
Triglycerides (mg/dL)	37 (80.43)	87.70	21.58	798.50	8 (17.40)	110.95	29.56	236.50	95.500	−1.559	0.11
HDL-C (mg/dL)	37 (80.43)	65.70	23.84	882.00	8 (17.40)	59.55	19.13	153.00	117.000	−0.921	0.35
**Glycemia**											
Fasting blood Glucose (mg/dL)	37 (80.43)	86.30	22.61	836.50	8 (17.40)	86.05	24.81	198.50	133.500	−0.431	0.66
**Total Physical Activity (min/wk)**	37 (80.43)	420.00	24.04	889.50	6 (13.04)	135.00	9.42	56.50	35.500	−2.648	0.008

Key: DHEA-S: dehydroepiandrosterone sulfate; Primary ALI: primary allostatic load index; PA: leisure sports; Total Physical Activity: sum of PA and exercise; Mdn: median; BMI: body mass index; HDL-C: high-density lipoprotein cholesterol; Significant at *p* < 0.05.

## Data Availability

Data containing potentially identified or sensitive patient information is restricted by European law (GDPR). The data used in this study involving clinical participants is unavailable in a public repository. However, data are available upon reasonable request to Pia-Maria Wippert (wippert@uni-potsdam.de).

## Abbreviations

The following abbreviations are used in this manuscript:

ACTH	Adrenocorticotropic Hormone
AL	Allostatic load
ALI	Allostatic Load Index
BMI	Body Mass Index
COMT	Catechol-O-Methyltransferase
CRH	Corticotropin-Releasing Hormone
DHEAS	Dehydroepiandrosterone sulfate
HDL-C	High-Density Lipoprotein Cholesterol
HIIT	High-Intensity Interval Training
HOMA	Homeostasis Model Assessment Index
HPA axis	Hypothalamic–Pituitary–Adrenal Axis
LBP	Low Back Pain
LC/NE	Locus Coeruleus/norepinephrine System
LDL-C	Low-Density Lipoprotein Cholesterol
MiSpEx	Medicine in Spine Exercise Network
PA	Physical Activity
PSA 3	Parallel Study 3
SNS	Sympathetic Nervous System
VAS	Visual Analog Scale
WHO	World Health Organization
